# Metoclopramide-induced acute dystonic reactions: a case report

**DOI:** 10.1186/s13256-021-03110-y

**Published:** 2021-10-24

**Authors:** Ritesh Chaudhary, Gyanendra Malla, Monika Kadayat

**Affiliations:** General Practice and Emergency Medicine, B.P Koirala Institute of Health Sciences, PO Box: 7053, Dharan, Nepal

**Keywords:** Acute dystonic reaction, Metoclopramide, Nepal

## Abstract

**Background:**

Acute dystonic reactions caused by drugs are uncommon in daily practice, whether in outpatient or in emergency settings. Such types of unfavorable reaction may cause the treating physician’s working diagnosis to be misled at a certain point.

**Case presentation:**

A 25-year-old Hindu female from Dharan with no previous medical history was being treated for acid peptic disease. Her local physician prescribed oral tablet metoclopramide 10 mg three times a day for 7 days and tablet pantoprazole 40 mg once daily for 7 days. After 24 hours of ingestion of 10 mg of tablet metoclopramide, she was admitted to our Koirala Institute of Health Sciences emergency department with sudden history of facial twitching, slurred speech, and abnormal tongue protrusion. Metoclopramide-induced acute dystonic reaction was diagnosed. After resuscitation, her symptoms reduced quickly, and she was successfully discharged home the same day.

**Conclusions:**

Early diagnosis would be aided by the use of clinical background along with focus on drug history usage, preventing life-threatening pitfalls. To decrease the acute dystonic reaction associated with metoclopramide use, higher frequency of prescription patterns should be taken into account.

## Introduction

Metoclopramide, an antiemetic medication, has been available in the USA since its approval by the Food and Drug Administration (FDA) in 1979. It is frequently used to treat esophageal reflux disease, dyspepsia, and gastroenterologic motility abnormalities, and to speed up stomach emptying time [1, 2]. Metoclopramide is a benzamide that acts as a dopamine receptor antagonist and is classified as a cholinomimetic (that is, increases the transmission of acetylcholine at muscarinic receptors) [3, 4].

The involuntary, prolonged, or spasmodic contractions of muscle groups that result in twisting, repeated, and aberrant postures are known as drug-induced acute dystonic reactions. If left untreated, such a problem can result in airway obstruction, temporomandibular joint dislocation, and oropharyngeal dysphagia [5]. When patients are administered metoclopramide or prochlorperazine, it has been observed that acute dystonic reaction occurs in 0.5–1% of cases [6]. Female patients, children, and individuals under the age of 30 years who received higher doses of metoclopramide had greater incidence [7]. Although many of the cases have been reported in Western countries, comparable occurrences have also been documented in Nepal and India [8, 9].

As a result, we present a similar and rare case of metoclopramide-induced acute dystonic reaction (MIADR) in the emergency department of a tertiary hospital in eastern Nepal.

## Case presentation

A 25-year-old unmarried Hindu female student from Dharan, Nepal, was brought to the emergency department of BPKIHS on 24 December 2019 at 4 pm with a 2 hour history of facial twitching, slurred speech, and 24 hour history of abnormal tongue protrusion. There was no documented history of any drug allergies or other abnormal body movements. She also did not have any relatives with seizure disorders. On 23 December, her local physician prescribed oral tablet metoclopramide 10 mg three times a day for 7 days and tablet pantoprazole 40 mg once every day for 7 days to prevent gastric acid and basal acid secretion for acid peptic disease (APD). On examination, her Glasgow Coma Scale (GCS) was 15, blood pressure 120/90 mmHg, pulse rate 102/min, and partial pressure oxygen saturation (SpO_2_) 99% on room air. Her arterial blood gas (ABG) revealed potential of hydrogen (P^H^)of 7.43, partial pressure of carbon dioxide (PCO_2_) at 31.1 mmHg; sodium ion (Na^+^) 140.3 mmol/L, potassium ion (K^+^) 3.87 mmol/L, concentration of hydrogen carbonate (HCO_3_^−^) 21.2 mmol/L. The Naranjo Adverse Drug Reaction Probability Scale scored 6 and classified as a “probable” adverse drug reaction [10]. Metoclopramide-induced acute dystonic reaction was diagnosed, and she was triaged as Australasian Triage Score (ATS) 2. She was resuscitated right away with intravenous fluids, oxygen via face mask, and a 25 mg intravenous dose of chlorpheniramine. After 2 hours, the symptoms subsided, and she was able to return home the same day. The patient was recommended to stop taking metoclopramide in the future.

## Discussion

Extrapyramidal adverse effects such as acute dystonia, tardive dyskinesia, akathisia, and drug‑induced Parkinsonism have been associated with the use of metoclopramide as an antiemetic.

Metoclopramide-induced acute dystonic reaction (MIADR) are found in 1:500 individuals [9], with a young female preponderance of up to 70% [7, 11], which was similar to our patient, a 25-year-old female, and could be a risk factor.

Following administration of metoclopramide, symptoms of MIADR may take up to 36 hours to appear, which is similar to our patient, who developed symptoms before 36 hours, which is comparable with another study [7].

According to the study, MIADR is thought to be associated with daily administration of more than 30 mg of metoclopramide [12]. However, MIADR was also observed even at the recommended dosage, which is comparable to our case. The patient was prescribed oral tablet metoclopramide 10 mg three times a day, which is similar to other studies reported from Nepal and India [8, 9].

Twitching of facial muscles, slurred speech, and aberrant rhythmic tongue protrusion are the most common symptoms of MIADR. However, throat discomfort and mild aphonia have also been reported as subtle presentation [7, 8, 13]. Other studies have also reported that these patients may present with combination of symptoms, including acute chorea [14], organic affective syndromes [15], and major depressive illness [16].

In the treatment of MIADR, diphenhydramine, anticholinergic benztropines are commonly used. Diazepam is utilized in refractory situations. Most of the signs and symptoms subsided after 5–15 minutes [17, 18]. Diphenhydramine and benztropine can improve the treatment of dystonia induced by antiemetics and psychotropic drugs [19].

Antihistamines are also effective for treating dystonia and reducing allergic manifestations [20, 21]. Intravenous chlorpheniramine 25 mg was used successfully to treat our patient.

MIADR is a rare emergency department presentation that can be easily overlooked or misinterpreted as conversion disorders or seizures. It can appear alone or in combination in normal prescribed dosages, posing serious challenges to diagnosis with a high chance of uncertainty.

## Conclusions

Emergency clinicians should be proficient in detecting MIADR effectively, allowing them to avoid potentially life-threatening situations. The greater frequency of metoclopramide prescription patterns, as well as the significant adverse dystonic reaction associated with its usage, should be taken into account.

## Data Availability

The datasets used and/or analyzed during the current study are available from the corresponding author on reasonable request.

## Abbreviations

APD: Acid peptic disease
ATS: Australasian Triage Score
ABG: Arterial blood gas
BPKIHS: BP Koirala Institute of Health Sciences
FDA: Food and Drug Administration
GCS: Glasgow Coma Scale
Hg: Mercury
mm: Millimeter
MIADR: Metoclopramide-induced acute dystonic reaction
mmol/L: Millimoles per liter
PCO_2_: Partial pressure of carbon dioxide
SPO_2_: Partial pressure oxygen saturation
P^H^: Potential of hydrogen
Na^+^: Sodium ion
K^+^: Potassium ion
HCO_3_: Concentration of hydrogen carbonate
